# Blunt Renal Trauma: A 6-Year Retrospective Review in a Single Institution

**DOI:** 10.3390/medicina61040621

**Published:** 2025-03-28

**Authors:** Anda Catinca Hogea, Ioan Scarneciu, Marius Alexandru Moga, Simona Grigorescu, Alina Bisoc, Mircea Daniel Hogea, Rosana Mihaela Manea

**Affiliations:** Faculty of Medicine, Transilvania University of Brasov, 500019 Brasov, Romania; anda.szasz@unitbv.ro (A.C.H.); ioan.scarneciu@unitbv.ro (I.S.); mogas@unitbv.ro (M.A.M.); simona.grigorescu@unitbv.ro (S.G.); alina.bisoc@unitbv.ro (A.B.); rosana.manea@unitbv.ro (R.M.M.)

**Keywords:** renal trauma, blunt injury, management, outcomes, retrospective study

## Abstract

*Background and Objectives*: Renal trauma is a significant consequence of both blunt and penetrating injuries, with management strategies having continuously evolved over recent years. This management requires careful clinical evaluation to balance the need for operative or non-operative treatment. This is especially critical in the context of the increasing trend of non-operative management for stable renal injuries, largely due to advances in imaging, improved hemodynamic stabilization, and better outcomes with conservative approaches. The main objectives of this study were to evaluate the epidemiology of renal trauma, the mechanism of injury, and the outcomes of management strategies in blunt renal trauma and determine their influence on morbidity and mortality rates. *Materials and Methods*: A retrospective review was conducted with patients diagnosed with renal trauma in the Emergency Clinical County Hospital in Brasov, Romania from 1.01.2018 to 31.12.2023. Data were collected from medical records. *Results*: A total of 89 patients with blunt renal trauma were identified. The most frequent renal injuries, according to AAST classification, were grade 2 in 34.83% of the patients and grade 1 in 26.97% of the patients. Most of them, 84.27%, were managed conservatively. The overall mortality rate was 12.36%. *Conclusions*: This review highlights the importance of personalized management strategies for renal trauma, especially emphasizing conservative treatment for hemodynamically stable patients. Our findings contribute to understanding renal trauma outcomes and should improve future clinical practices and guidelines in renal trauma management. Further studies should explore long-term outcomes and optimize treatment protocols.

## 1. Introduction

Renal trauma is a prevalent injury in the context of both blunt and penetrating trauma, often resulting from incidents such as motor vehicle accidents, falls, sports injuries, and assaults. Blunt renal trauma (BRT) accounts for approximately 80–90% of all renal injuries, making it the most common form of kidney trauma. The kidneys are particularly vulnerable due to their location and the nature of these traumatic events. Understanding the incidence, management strategies, and outcomes associated with renal injuries is crucial for optimizing patient care. Understanding the mechanisms, classification, and management of BRT is essential for trauma surgeons, urologists, and emergency physicians to optimize patient care and prevent long-term renal complications [1,2,3,4].

Renal trauma refers to kidney injuries due to blunt or penetrating forces, often resulting from motor vehicle accidents, falls, or violent acts. Kidney injuries are a significant cause of morbidity and sometimes mortality, especially in patients with associated trauma to other organs [5].

The American Association for the Surgery of Trauma (AAST) (Table 1) classifies renal injuries into five grades, ranging from minor contusions to major lacerations and vascular injuries. This classification contributes to determining the most suitable management approach [6,7,8,9].

Historically, surgical intervention has been the “golden” standard for higher-grade injuries; however, there has been a shift toward conservative management for select cases, particularly for lower-grade injuries in stable patients. Non-operative management often involves careful monitoring and supportive care, reducing the need for invasive procedures and associated complications [5,10].

The clinical aspects of renal trauma can vary significantly. While hematuria and flank pain are common symptoms, in some patients, few signs can occur until complications arise. Imaging studies, particularly CT scans, are essential for the accurate diagnosis and assessment of injury severity, guiding post-traumatic management decisions. The advent of contrast-enhanced computed tomography (CT) with delayed excretory phases has revolutionized the diagnosis and grading of blunt renal trauma. This modality allows for detailed assessment of renal parenchymal damage, vascular integrity, and urinary extravasation, helping to guide management decisions [11,12,13,14].

Outcomes following renal trauma can be favorable, with many patients experiencing good recovery. Blunt renal trauma remains a clinically significant and evolving field in trauma care. While non-operative management has become the standard of care for stable patients, high-grade injuries still pose challenges in diagnosis, intervention, and long-term follow-up [1].

This retrospective review aims to analyze renal trauma cases at the Emergency Clinical County Hospital in Brasov, Romania, over a six-year period, focusing on incidence, management strategies, and clinical outcomes. By examining local practices and trends, this study seeks to enhance understanding of renal trauma management and improve future protocols to improve patient outcomes.

## 2. Materials and Methods

### 2.1. Study Design

This study is a retrospective cohort analysis conducted at the Emergency Clinical County Hospital in Brasov, Romania. It reviews all cases of nonpenetrating renal trauma over a six-year period from 1 January 2018 to 31 December 2023.

This research did not involve any risk for the participants. All ethical guidelines were followed as required for conducting human research. The procedures performed in this study involving human participants were in accordance with the ethical standards of the institutional research, with this study being approved to be run by the Ethical Commission of the Emergency County Hospital in Brasov, where this study was conducted, and by the Committee for Ethical Research of Transylvania University. This research was also consistent with the ethical guidelines of the College of Physicians in Romania. This research also complies with the provisions of the Declaration of Helsinki (as revised in Brazil in 2013). The anonymity of all the participants was carefully and absolutely preserved.

### 2.2. Participants

All patients diagnosed with blunt renal trauma during the study period were included.

### 2.3. Data Collection

Data were extracted from electronic medical records and included:

- **Demographics**: Age, gender, environment (rural/urban);
- **Mechanism/Incidence of injury**: Falls, traffic accidents, aggression;
- **Injury Severity**: Classification of renal injuries using the AAST grading system (grades 1 to 5);
- **Clinical Aspects**: Symptoms and signs at presentation, including hematuria, flank pain, and signs of shock;
- **Imagistic studies (ECO, CT**–GE Optima 660, 128 slices);
- **Blood tests**;
- **Management Strategies**: Details on the management approach (conservative vs. surgical);
- **Outcomes**: Post-treatment outcomes and mortality rates.

Statistical analysis was conducted using MedCalc 9.2.1.0 and Microsoft Excel 2021.

This methodology aimed to provide a comprehensive overview of blunt renal trauma cases, facilitating insights into effective management strategies and patient outcomes within the institution.

## 3. Results

### 3.1. Demographics

**Figure 1:**
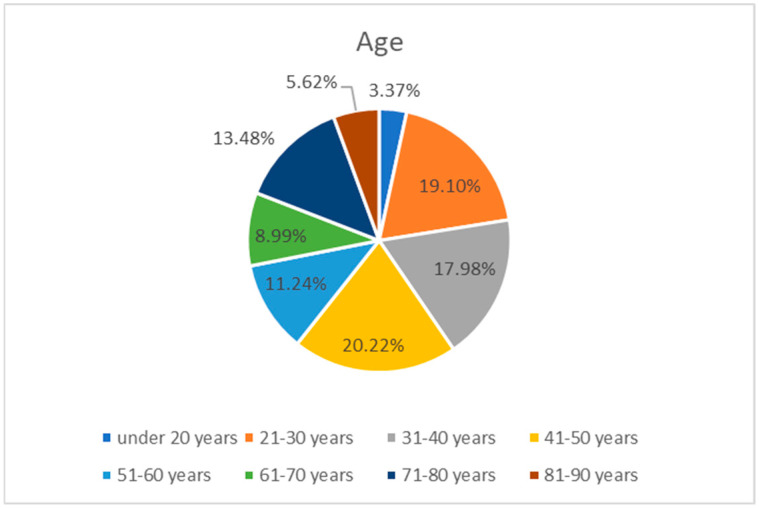
Age distribution.

A total of 89 patients with blunt renal trauma were identified during the study period. Their ages varied from 20 to 86 years, with a mean age of 47.87 years. The highest incidence of renal trauma was between 41 and 50 years. (20.22%), followed by the group between 21 and 30 years (19.1%) (Figure 1). The standard error of the mean (2.06 years) suggests a precise estimate of the population mean, but a larger sample could further reduce uncertainty. The skewness (0.3642, *p* = 0.1492) suggests a slight right skew (more older individuals), but it is not statistically significant. The kurtosis (−1.0534, *p* = 0.0765) indicates a flatter distribution (fewer extreme values) than a normal curve, though not significantly different from normal (Table 2).

**Figure 2:**
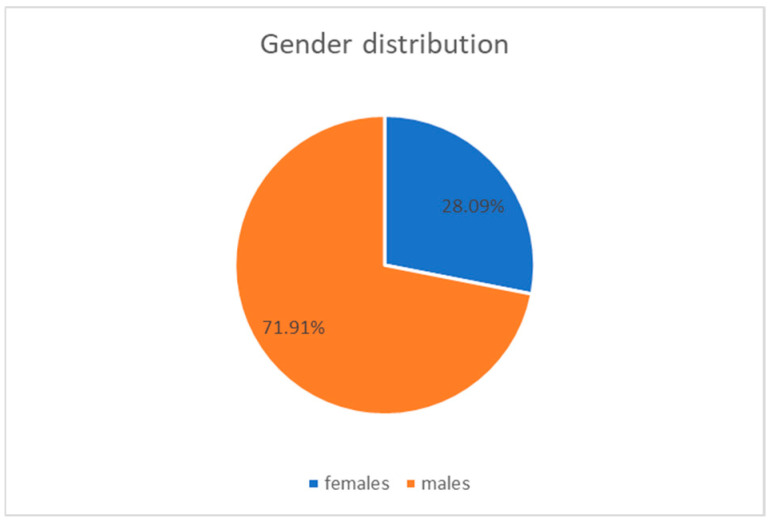
Gender distribution.

Most of the patients with blunt renal trauma were males (71.91%), with 28.09% females (Figure 2). The most frequent type of injury was falls, in 51.69% of the cases, followed by traffic accidents in 33.71% and aggression in 14.61%.

#### 3.1.1. Mechanism/Incidence of Injury

**Figure 3:**
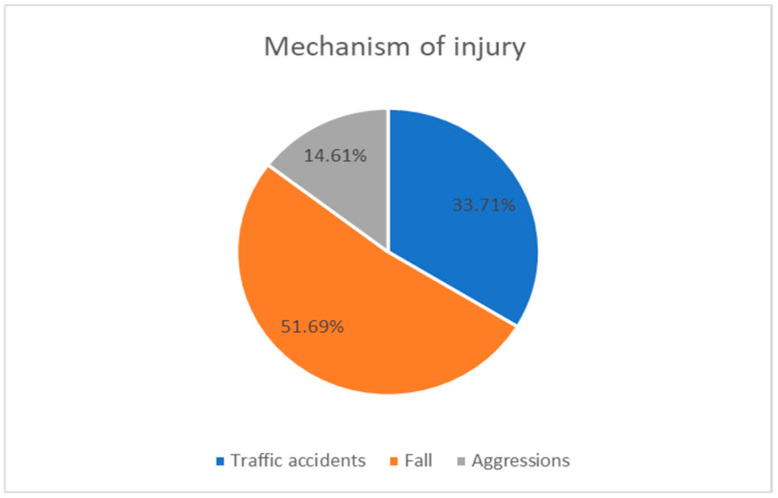
Mechanism of injury distribution.

In the context of kidney trauma, most injuries were due to falls (51.69%), followed by traffic accidents (33.71%) and aggression (14.61%) (Figure 3).

#### 3.1.2. Six-Year Incidence of Kidney Trauma (From 2018 to 2023)

**Figure 4:**
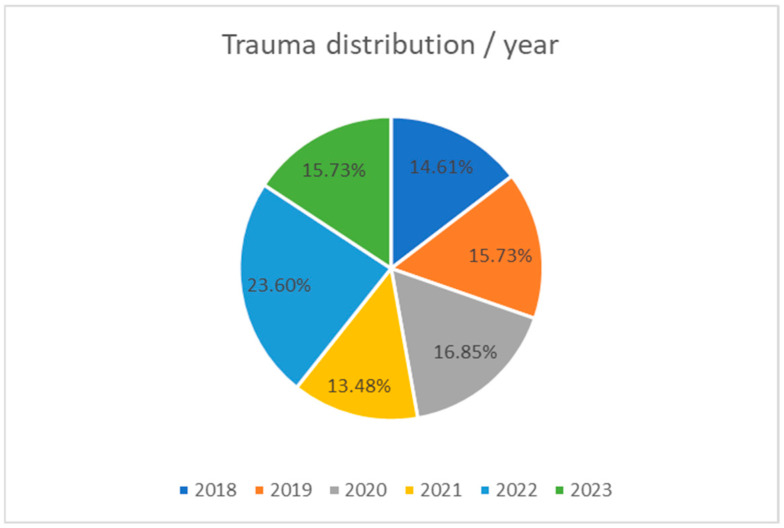
Trauma distribution/year.

In all six years, we had similar numbers of patients, with a peak in 2022 (Figure 4):

- 2018: 13 patients (14.61%);
- 2019: 14 patients (15.73%);
- 2020: 15 patients (16.85%);
- 2021: 12 patients (13.48%);
- 2022: 21 patients (23.6%);
- 2023: 14 patients (15.73%).

### 3.2. Injury Severity

**Figure 5:**
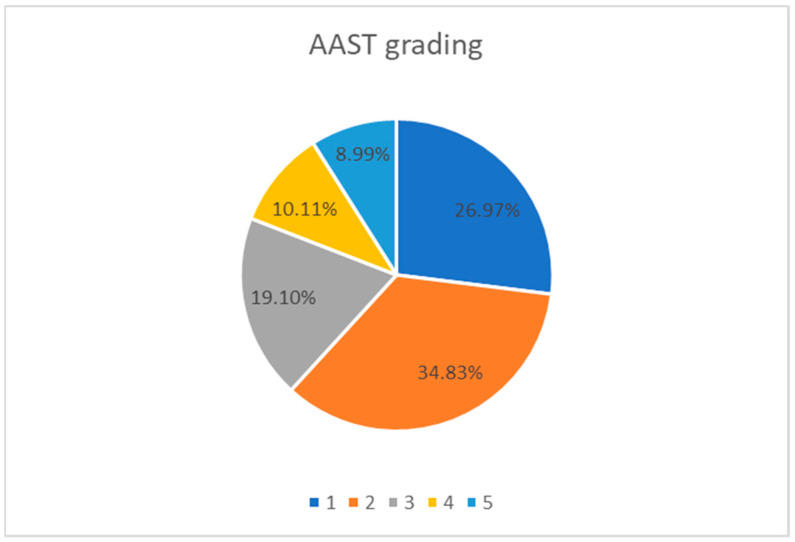
AAST grading distribution.

**Figure 6:**
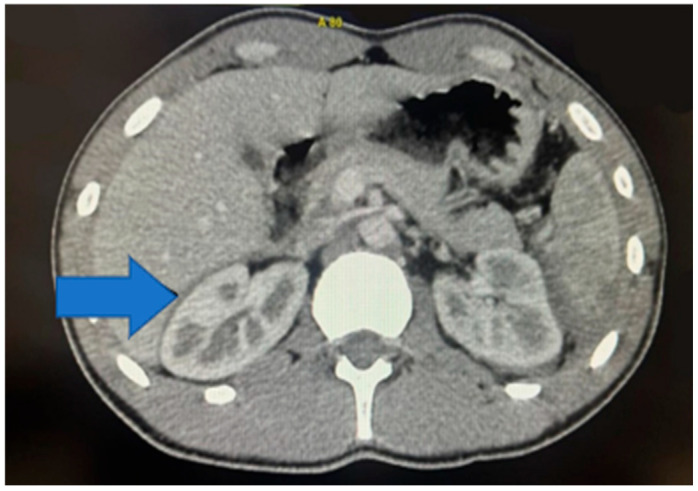
Contrast-enhanced CT scan showing right-kidney grade 1 injury—minimum hematoma.

**Figure 7:**
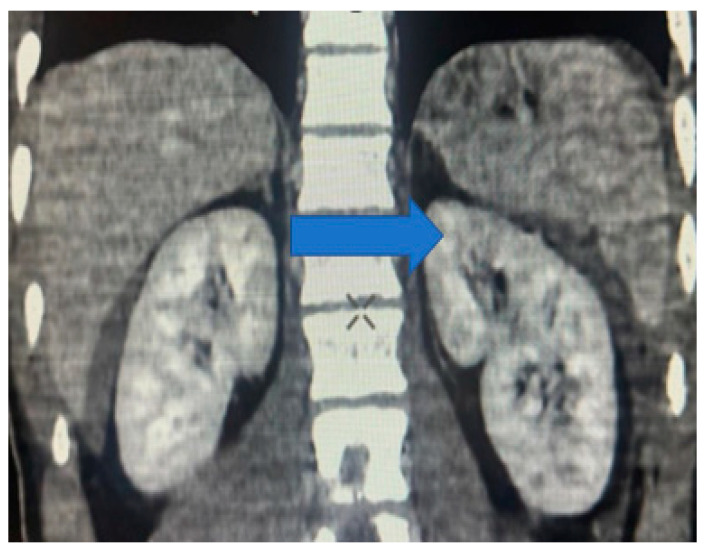
Contrast-enhanced CT scan showing left-kidney grade 2 injury—hematoma of < 1 cm depth.

**Figure 8:**
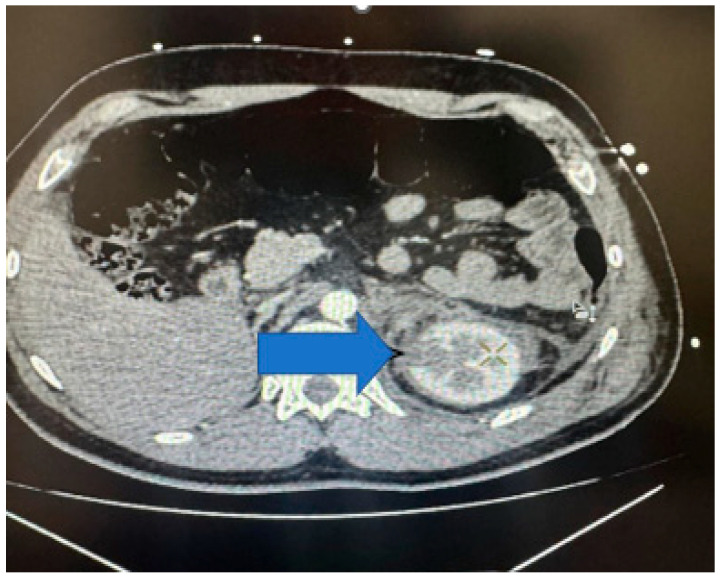
Contrast-enhanced CT scan showing left-kidney grade 3 injury—laceration of >1 cm.

**Figure 9:**
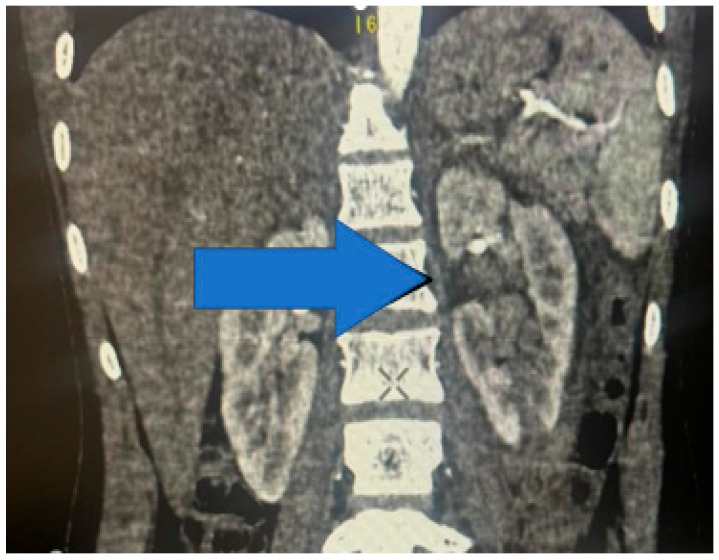
Contrast-enhanced CT scan showing left-kidney grade 4 injury—parenchymal laceration.

**Figure 10:**
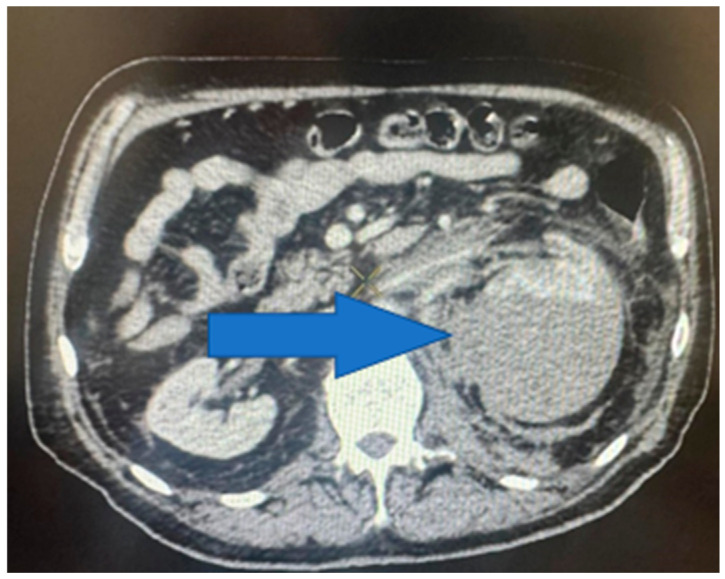
Contrast-enhanced CT scan showing left-kidney grade 5 injury—avulsion of renal hilum with large hematoma, axial section.

**Figure 11:**
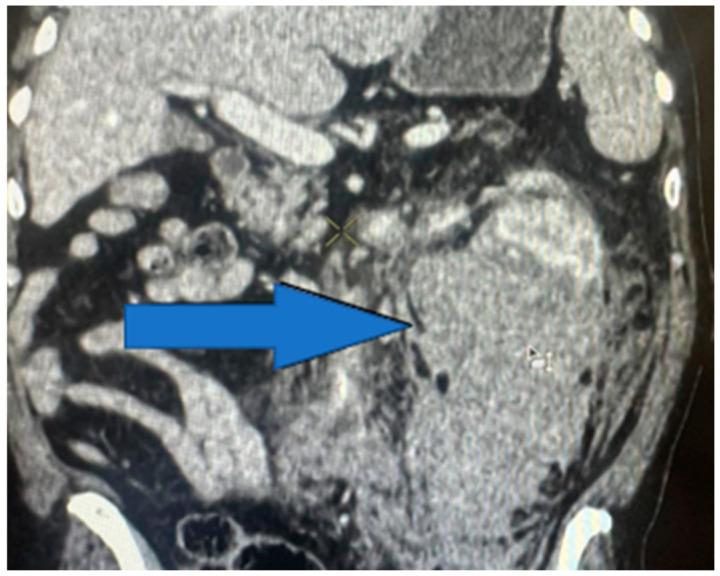
Contrast-enhanced CT scan showing left-kidney grade 5 injury—avulsion of renal hilum with large hematoma, coronal section.

The distribution of the renal injuries, according to AAST classification (Figure 5), in this study is the following: grade 1 (Figure 6) (26.97%), grade 2 (Figure 7) (34.83%), grade 3 (Figure 8) (19.10%), grade 4 (Figure 9) (10.11%), and grade 5 (Figure 10 and Figure 11) (8.99%).

The logistic regression model indicates that AAST classification has a strong and statistically significant effect on the likelihood of receiving treatment (*p* = 0.0001). The positive coefficient (2.3260) suggests that higher AAST values increase the probability of treatment. The odds of receiving treatment increase 10.24 times for each unit increase in AAST classification (Odds Ratio = 10.2373). The confidence interval (3.3192 to 31.5745) does not include 1, confirming statistical significance. The chi-square test (45.7659, *p* < 0.0001) indicates that this model provides a significantly better fit than a null model (Table 3).

#### The Side Prevalence of Affected Kidneys

**Figure 12:**
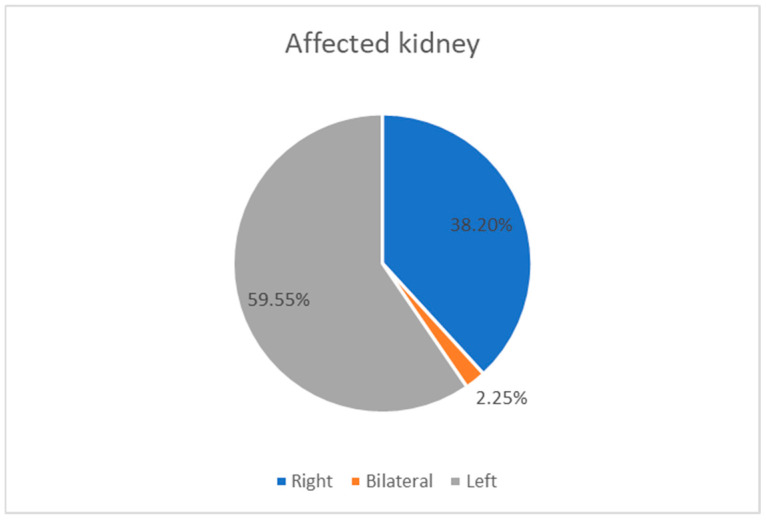
Affected kidneys.

The most affected kidney (referring to the right/left side) was the left—53 patients, (59.55%). The right kidney was affected in 34 patients (38.2%), and in 2 cases (2.25%), both kidneys were affected (Figure 12).

### 3.3. Clinical Aspects

**Figure 13:**
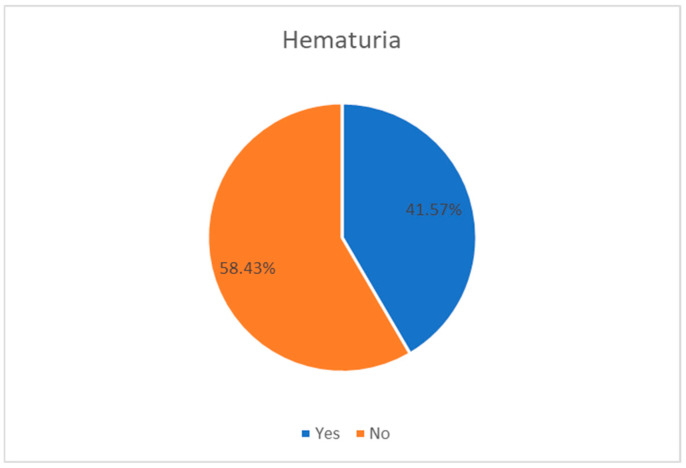
Presence of hematuria.

**Figure 14:**
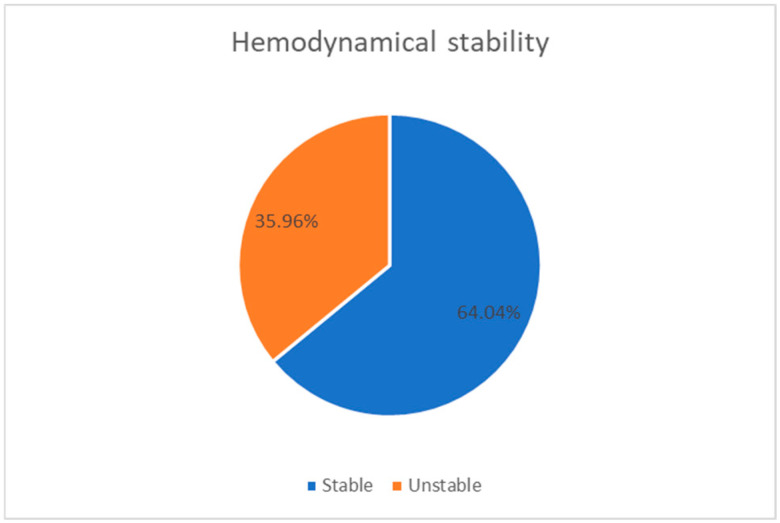
Hemodynamical stability distribution.

At presentation, all patients reported flank pain, 41.57% exhibited hematuria (Figure 13), and 36% presented hemodynamic instability signs (Figure 14). The mean arterial pressure was between 46.67 and 139.33 mmHg (Table 4), and the pulse varied from 50 to 123 bpm with a mean heart rate of 84.4 bpm.

The near-zero skewness suggests the data are almost symmetrical. The positive kurtosis suggests a slight tendency toward a more peaked distribution than normal. The dataset appears normally distributed based on skewness, kurtosis, and the chi-square test. Since the SEM (standard error of the mean) = 1.8087, the sample mean (87.87) was estimated with an error margin of ±1.8087 (Table 4).

The positive skewness coefficient suggests a slight rightward tail, and the positive kurtosis suggests a distribution that is slightly more peaked than normal. The heart rate data follow a normal distribution, as indicated by the normality tests. The standard deviation (14.83) suggests moderate variability in heart rate. The mean and median are close, further indicating a roughly symmetrical distribution. Since the SEM = 1.5809, the mean heart rate (84.41 bpm) was estimated with an error margin of ±1.5809 (Table 5).

**Figure 15:**
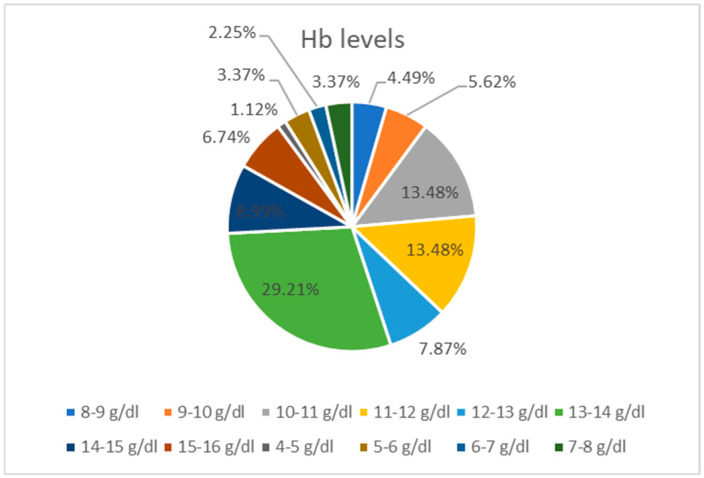
Hemoglobin level distribution.

The most patients had, at the moment of presentation, a level of hemoglobin higher than 12 g/dL (47 patients, 52.80%) (Figure 15).

**Figure 16:**
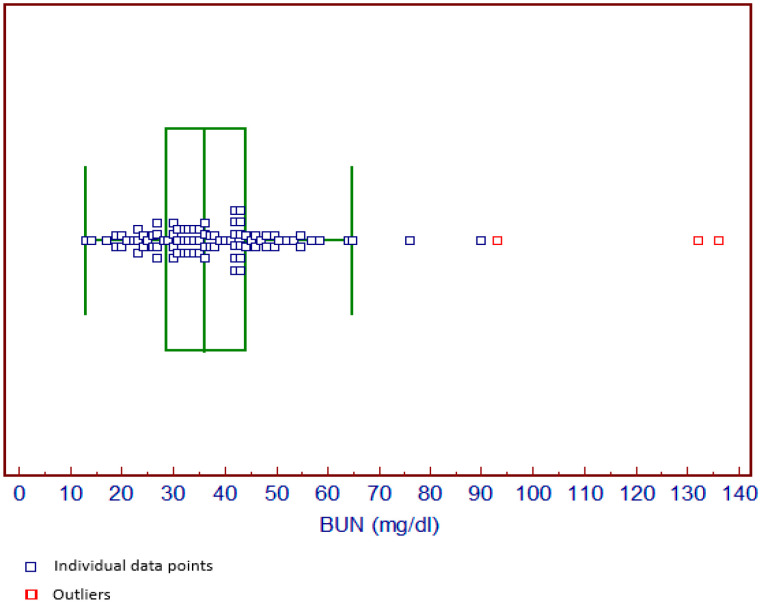
Boxplot showing the distribution of BUN (mg/dL).

The lowest value of blood urea nitrogen (BUN) was 13.0 mg/dL, and the highest value was 136.1 mg/dL. The positive skewness (2.65) means the data distribution is right-skewed, with a longer tail toward higher BUN values (Figure 16). The high kurtosis (9.71) suggests a leptokurtic distribution, meaning more extreme values (outliers) than a normal distribution. SEM = 2.1437 (Table 6).

**Figure 17:**
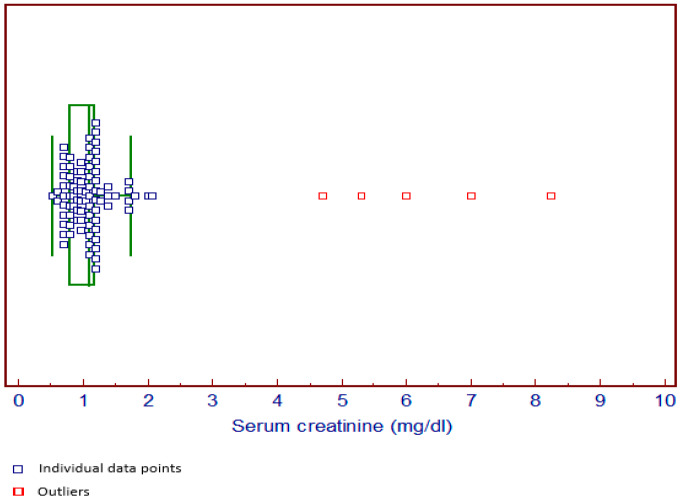
Boxplot showing the distribution of serum creatinine (mg/dL).

The lowest creatinine level value was 0.53 mg/dL, and the highest value was 8.23 mg/dL. The positive skewness (3.93) means the distribution is highly right-skewed, with extreme values on the higher end (Figure 17). The very high kurtosis (15.81) suggests a leptokurtic distribution, meaning many extreme values (outliers) compared with normal distribution. SEM = 0.1350 (Table 7).

#### 3.3.1. Previous Renal Pathology

**Figure 18:**
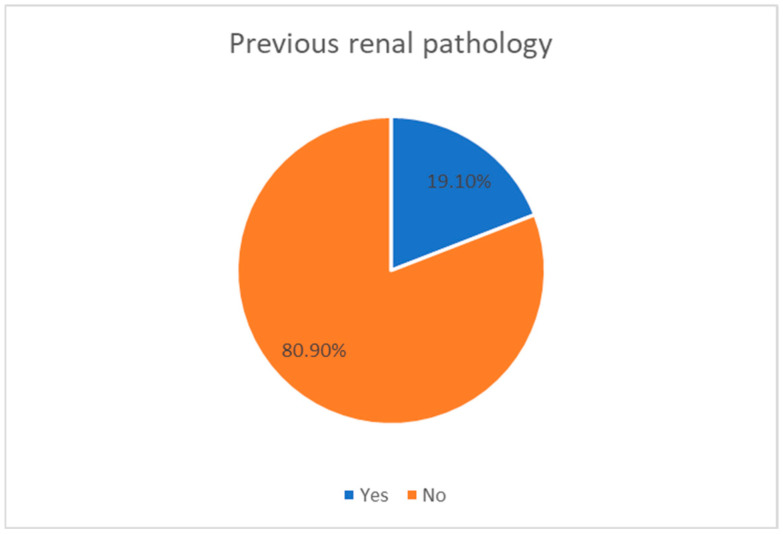
Previous renal pathology.

The incidence of previous renal pathology in the patients with renal trauma was 19.10% (Figure 18). We found most to have numerous abnormalities like cysts, hydronephrosis, and malposition. Also, we found five patients with kidney failure.

#### 3.3.2. Associated Organ Injury

**Figure 19:**
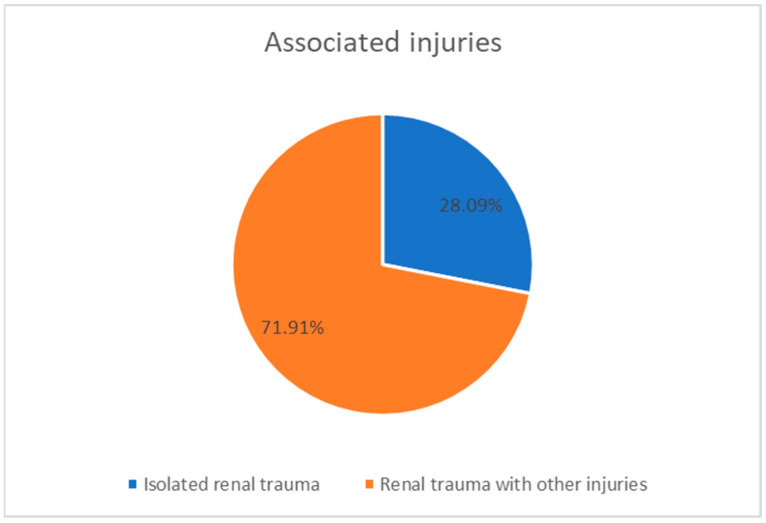
Associated injuries.

**Figure 20:**
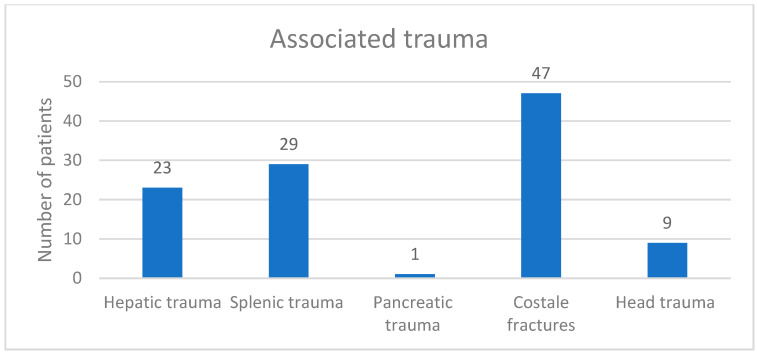
Associated trauma.

The majority of the patients with renal trauma had associated injuries (71.91%) (Figure 19). The most associated injuries were costal fractures (47 patients), followed by splenic injury (29 patients) and hepatic trauma (23 patients). Only 28.09% of the patients presented isolated renal trauma (Figure 20).

### 3.4. Management Strategies

**Figure 21:**
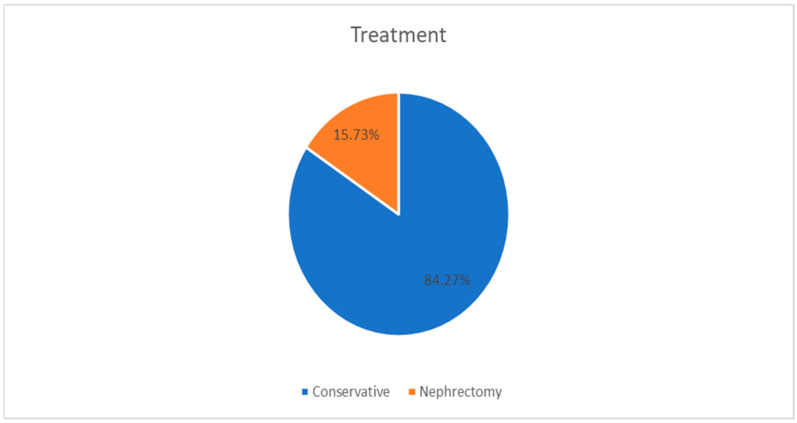
Treatment management.

The management of the renal trauma was categorized as follows: 75 patients (84.27%) were treated in a conservative manner, and 14 patients (15.73%) underwent surgical procedures finalized by nephrectomy (Figure 21).

#### Length of Hospital Stay

**Figure 22:**
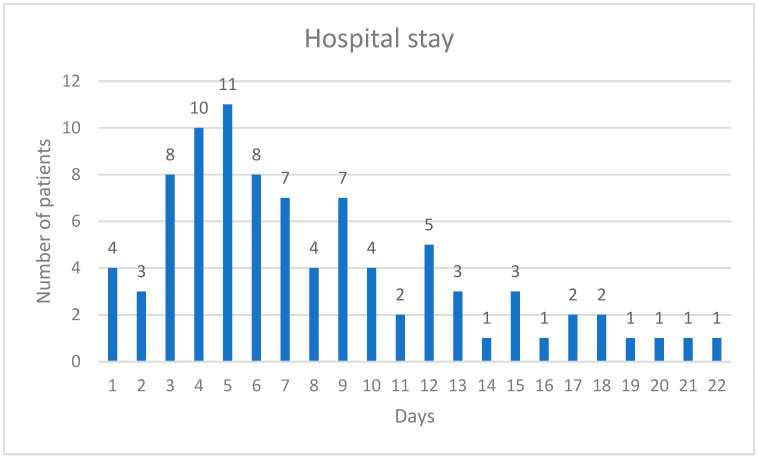
Length of hospitalization (days).

The longest period of hospitalization was 35 days, and the shortest was 1 day, with a mean hospital stay length of 8.37 days (Figure 22).

The positive skewness indicates a long right tail (some patients had much longer stays). The high kurtosis suggests heavy tails (more extreme values than a normal distribution). Since the SEM = 0.6494, the mean hospital length of stay (8.37 days) was estimated with an error margin of ±0.6494 (Table 8).

### 3.5. Outcomes

**Figure 23:**
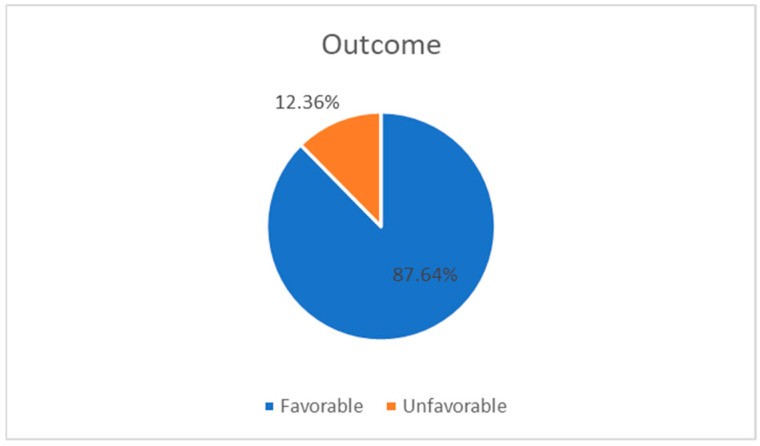
Clinical outcome.

The overall mortality rate was 12.36%. All of the deceased patients presented multiple-organ high-grade associated injuries (Figure 23).

The overall mortality rate in the patients managed non-operatively was 9.33%. All of them had lower grade renal injury, but had also associated high-grade injuries involving other organs (spleen, liver).

The overall mortality rate in the patients after nephrectomy was 28.57%. All of them presented high-grade renal injury in association with high-grade injuries involving other organs. All these patients were diagnosed in the emergency room with hemodynamic instability.

## 4. Discussion

This study provides a comprehensive analysis of blunt renal trauma cases over a six-year period at the Emergency Clinical County Hospital in Brasov, Romania, highlighting key aspects of incidence, treatment management strategies, and clinical outcomes. These findings contribute to the growing body of the literature on renal trauma and underscore the importance of personalized treatment approaches.

The number of patients over the studied period was almost constant during each year (12–15 patients/year), with a peak in 2022 (reaching 21 patients).

The male-to-female ratio for renal trauma tends to favor males. This means that males are generally more exposed to experience renal trauma than females. Several factors contribute to this, but the most important is the higher exposure to risk factors; males are often more involved in activities that increase the risk of trauma, such as contact sports, motor vehicle accidents, and violent altercations [2,15,16,17,18,19]. The demographic profile of this cohort has a predominance of males: 71.91%. In this study, the highest incidence was in the 41–50 age group.

Blunt trauma is the most common cause of renal injuries. Blunt injuries are caused by forces that impact the body without penetrating the skin. In this study, injury from falling was the most frequent mechanism, despite the trend shown in the literature where the most frequent cause of injury is motor vehicle accidents [16,18].

In renal trauma, both the left and right kidney can be affected, but there are some variations in how often each side is injured based on factors like anatomical position and mechanism of injury [1,12]. In our study, the left kidney was affected in 59.55% of the cases.

Based on several studies and trauma reviews, isolated renal trauma is less common than renal trauma with associated injuries [1]. In this study, most patients with renal trauma had associated injuries (71.91%). Also, in most of the studies cited in the literature, the patients with renal trauma had other associated injuries [16].

Hematuria is a common clinical sign of renal trauma, and it is used as a diagnostic indicator in patients with suspected kidney injuries. Hematuria is often one of the earliest signs of kidney trauma. It can appear immediately after injury, making it a rapid and sensitive indicator of renal damage. The presence of red blood cells in the urine can suggest damage to the renal parenchyma, blood vessels, or urinary tract. The relationship between hematuria and renal trauma is often used to help guide further diagnostic steps and treatment decisions [2,3,12,17,20]. In our study, we had 41.57% of patients with hematuria at the presentation time.

Kidney function tests (such as serum creatinine and blood urea nitrogen) can remain within normal ranges in the early stages of kidney injury, as the unaffected kidney will compensate for the damage. Extreme levels of creatinine (over 2.5 mg/dL) and blood urea nitrogen (65 mg/dL) were found in the patients with previous kidney failure.

Our results demonstrated a wide distribution of injury severity, with the majority classified as lower-grade injuries (grades 1–3). The majority of the renal trauma in this study was grade 2 (AAST score)—34.83%, followed by grade 1—26.97%.

The logistic regression analysis provides strong evidence that AAST classification is a significant predictor of treatment. This model demonstrates high accuracy and effectively differentiates between cases. Given its strong performance, it could be a useful tool for predicting treatment decisions based on AAST values.

The conservative treatment was the most frequent type of treatment applied to the patients in this study (83.15%). This is also the trend observed in many studies in the literature [16,21,22]. The trauma management according to the AAST classification was as follows: With grade 1 and grade 2, all patients were treated conservatively. Nephrectomy was performed in three cases of grade 3 renal trauma and also in three cases of grade 4 renal trauma. All patients with severe renal trauma, grade 5, were treated nonconservatively. All these data are similar with those found in the literature, where the studies showed that grade 5 renal trauma is treated operatively. The solution for successfully avoiding nephrectomy in these high-grade patients is angioembolization [3,11,13,17,23].

The nephrectomy rate in this study was 15.73%, similar to other studies [21,24,25].

This finding supports the trend toward conservative management for lower-grade renal injuries, which has been increasingly adopted in clinical practice [1,26,27]. The significant proportion of patients treated non-operatively underlines the efficacy of careful monitoring and supportive care in stable patients. Conversely, the necessity for surgical intervention in higher-grade injuries is consistent with established guidelines [1,8,28].

The incidence of pre-existing renal pathology in patients with renal trauma is a relevant factor in the evaluation and management of kidney injuries. Pre-existing renal conditions may influence the severity of trauma, the patient’s ability to recover, and the treatment approach. Some studies correlate pre-existing renal pathology with a high risk of non-operative management failure [3,29,30]. In this study, 19.10% of the patients had pre-existing renal pathology (cysts, hydronephrosis, malposition, kidney failure) or renal complications generated by diabetes mellitus, primary or secondary arterial hypertension, or certain forms of chronic nephritis [31]. The nephrectomy rate among these patients was 35.29%.

The length of hospitalization for renal trauma patients can vary significantly depending on factors such as the severity of the injury, the presence of associated injuries, the treatment approach (conservative vs. nonconservative), and the patients’ comorbidities. In the literature, hospitalization days for renal trauma are typically categorized based on injury severity and treatment modality [32]. In this study, the mean hospital stay was 8.37 days.

The mortality rates in patients with renal trauma can vary depending on several factors, including the severity of the trauma, the presence of associated injuries, the mechanism of injury and the timelines of treatment. In this study, the mortality rate was 12.36%, being generally lower in cases of isolated renal trauma. We found that it can increase significantly when there are associated injuries such as vascular injuries, trauma to other organs, or significant hemorrhage. The presence of chronic immune diseases also affecting the renal system seems to increase the risk of mortality [1,33,34].

One of the main concerns of traumatologists, which could explain the primary tendency to choose the conservative treatment strategy over the primary surgical approach, is the possibility of reaching a correct and complete diagnosis as fast as possible in order to ensure a better and personalized strategy of treatment.

**Scheme 1:**
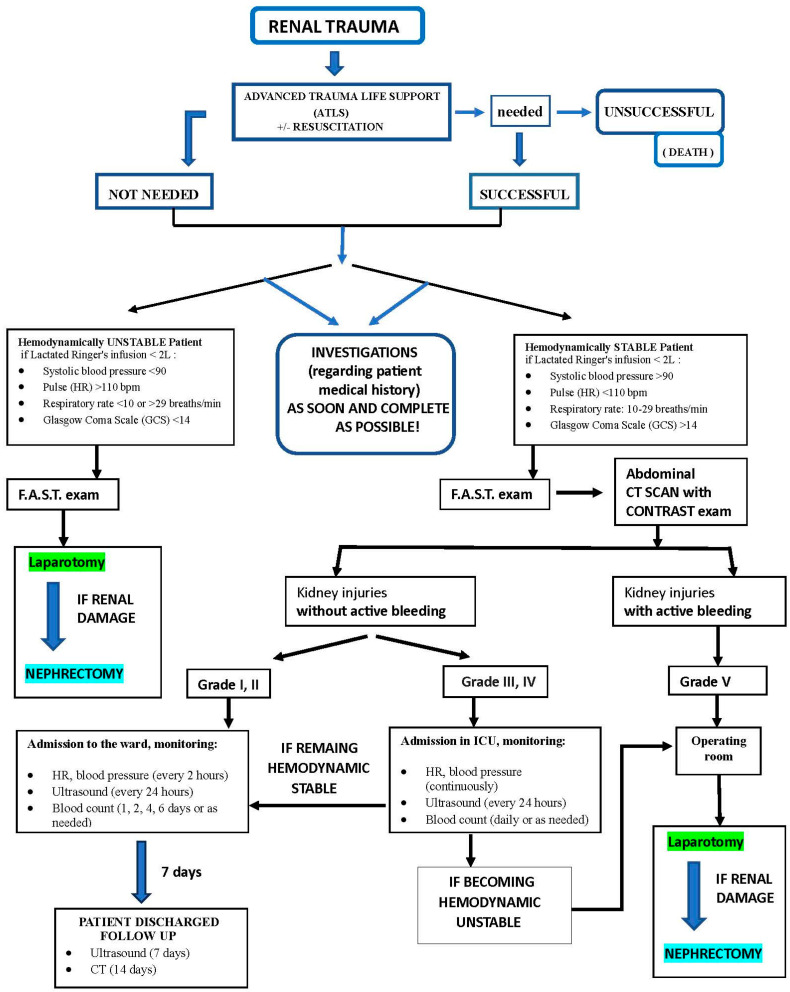
Algorithm for renal trauma management.

In this context, we propose a new and clear algorithm regarding the way in which patients with renal trauma will be managed (Scheme 1). The proposed algorithm would be able to provide a rigorous approach to all kidney trauma, which could be followed in order to obtain optimal results.

The first stage of the new proposed algorithm for renal trauma management is to determine if performing advanced trauma life support (A.T.L.S.) and resuscitation procedures is needed for certain. A.T.L.S. includes airway stabilization, breathing assessment, circulation support, and immediate resuscitation measures such as fluid replacement and blood transfusion. If performing A.T.L.S. and resuscitation procedures is unsuccessful and the patient does not survive, from that moment, the algorithm is not useful and must stop. If the A.T.L.S. procedures are successful, sustainable blood pressure and heart and respiratory rates will be generated, allowing passage to the second step of the algorithm, as well as if A.T.L.S. and resuscitation procedures are not needed.

The second stage of the algorithm involves assessment of the hemodynamic stability of the patient.

The category of hemodynamically unstable patients is characterized by some principal symptoms, such as a systolic blood pressure of below 90 mmHg, a heart rate of above 110 beats per minute, abnormal respiratory rates, and a Glasgow Coma Scale (GCS) score of below 14. An immediate Focused Assessment with Sonography for Trauma (F.A.S.T.) examination in order to assess internal bleeding is required as soon as possible for these patients. If any renal damage is detected as a source of bleeding, an urgent laparotomy must be performed. A nephrectomy may be the only solution for patient survival.

The other category, of hemodynamically stable patients, has as its main characteristics a systolic blood pressure above 90 mmHg, a heart rate between 50 and 100 beats per minute, and a respiratory rate of 12–20 breaths per minute. These patients also mandatorily undergo F.A.S.T. examinations followed by abdominal CT scans with contrast, which could provide more detailed evaluation of renal injuries.

At the same time that the hemodynamic component is evaluated, patients from both previously mentioned categories must be mandatorily investigated regarding all their medical history to put into evidence the chronic diseases (diabetes, high blood pressure, atrial fibrillation, etc.) that could negatively affect the evolution of new renal lesions.

The third stage of the algorithm depends on the existence of active intra-abdominal bleeding and consists of choosing the most suitable way to approach patients with renal injuries classified in grades 1–5 of renal injury.

Kidney injuries with active bleeding are included in the 5th-grade renal injuries. The patients with this severe condition are critical and often require emergency surgery. Each patient is sent directly to the operating room for laparotomy. If the renal damage is significant, a nephrectomy is performed in order to take control of the bleeding and prevent further complications.

In the cases of patients with kidney injuries without active bleeding (classified into grades 1–4 based on severity condition), the algorithm proposes for those with grade 1 and 2 (minor kidney injuries without active bleeding) to be admitted to the ward for continuous monitoring of heart rate, blood pressure, and blood count. Ultrasounds are performed every 24 h, and blood counts are checked periodically (days 1, 2, 4, and 6 and as needed).

In the cases of patients with moderate to severe kidney injuries and also without active bleeding (grade 3 and 4 renal injuries), admission to the Intensive Care Unit (ICU) for closer monitoring is mandatorily required. For this category of patients, heart rates and blood pressure are continuously monitored, ultrasounds are performed every 24 h, and blood counts are checked daily. If a patient remains stable, no surgical intervention is needed.

The fourth stage of the algorithm is activated if a patient that managed to evolve hemodynamic stability becomes subsequently hemodynamically unstable. In this case, if instability develops, a laparotomy must be performed, and nephrectomy becomes a procedure to be seriously taken into consideration.

For the patients who remain stable and, therefore, do not require nephrectomy, follow-up care is crucial. If no complications arise within seven days, nephrectomy is definitely avoided. All discharged patients undergo ultrasound check-ups on day 7 and CT scans at 14 days, as continuous checking of the renal function ensures the best conditions for recovery for the patients.

In conclusion, regarding the new algorithm, renal trauma management requires a systematic approach beginning with resuscitation and assessment of hemodynamic stability. Based on the severity of injury, patients undergo different diagnostic and treatment pathways. While mild to moderate injuries can often be managed conservatively with close monitoring—the vital signs monitored closely are heart rate, blood pressure, urine output, and IV fluids to maintain normovolemia—and crystalloids are preferred, as well as blood transfusion as needed for anemia (Hb < 7–8 g/dL), severe injuries necessitate surgical approaches, including nephrectomy. Proper and rigorous follow-up is essential to ensure full recovery and prevent long-term complications (initial CT with contrast for staging of renal injury; repeat imaging in 24 h for patients with high-grade injuries, persistent hematuria, or worsening clinical status). Pharmacological management includes pain control (acetaminophen, opioids if needed), antibiotics in cases of urinary extravasation to prevent urosepsis, and antihypertensive therapy for post-traumatic renovascular hypertension. By following structured clinical guidelines or algorithms, as healthcare providers, we can improve survival rates and optimize patient care in renal trauma.

These results underscore the importance of individualized management strategies for renal trauma, highlighting the effectiveness of conservative treatment for lower-grade injuries while noting the challenges associated with higher-grade injuries requiring surgical intervention [1,27]. 

For more than two decades, the management of renal trauma has been a controversial subject regarding non-operative versus operative management, with guidelines recommending the initiation of non-operative management in all patients as long as they were hemodynamically stable [19,26,29].

### Limitations

This study has several limitations. The retrospective design may have introduced biases related to data collection and patient selection. Additionally, the single-institution setting may have limited the accuracy of the findings. Further, the sample size may not have captured all variations in management strategies or outcomes, particularly in rare cases.

## 5. Conclusions and Future Directions

In conclusion, this study highlights the importance of personalized management strategies for renal trauma. Our findings support the continued use of conservative management for stable patients while emphasizing the need for careful decision-making regarding surgical interventions for higher-grade injuries.

Renal trauma is a complex and potentially life-threatening condition that requires prompt and complete diagnosis followed by appropriate management. Using a clear algorithm, as the one proposed in our study, must be determinant for choosing the best therapeutic strategy. The early imaging and classification of renal injuries play crucial roles in guiding treatment. Conservative management is often effective for grade 1–3 injuries, while more severe injuries may require surgical intervention. The presence of associated injuries, pre-existing renal pathology, and the mechanism type of the injury significantly influence outcomes. Advances in minimally invasive techniques and early detection are likely to improve both short-term recovery and long-term kidney preservation for patients with renal trauma.

Further research is needed to refine non-invasive diagnostic techniques (such as advanced imaging modalities) and to explore the role of embolization and other minimally invasive techniques in managing high-grade renal trauma.

There is a need for large multicenter studies to better understand the long-term outcomes of renal trauma patients, especially those with pre-existing renal conditions, to optimize management protocols.

## Figures and Tables

**Table 1:** AAST classification.

Grade	Type	Description
I	Contusion	Microscopic or gross hematuria. Urological studies normal.
	Hematoma	Subcapsular, non-expanding without parenchymal laceration.
II	Hematoma	Non-expanding peri-renal hematoma confined to renal retroperitoneum.
	Laceration	<1.0 cm parenchymal depth of renal cortex with no urinary extravasation.
III	Laceration	>1.0 cm parenchymal depth of renal cortex without collecting system rupture or urinary extravasation.
IV	Laceration	Parenchymal laceration extending through renal cortex, medulla & collecting system.
	Vascular	Main renal artery or vein injury with contained hemorrhage.
V	Laceration	Completely shattered kidney.
	Vascular	Avulsion of renal hilum that devascularises kidney.

**Table 2:** Distribution of patients by age (years).

Variable	Age (years)
Lowest value	20.0000
Highest value	86.0000
Arithmetic mean	47.8764
95% CI for the mean	43.7761 to 51.9767
Median	45.0000
95% CI for the median	38.0000 to 51.1038
Variance	378.8823
Standard deviation	19.4649
Standard error of the mean	2.0633
Coefficient of Skewness	0.3642 (*p* = 0.1492)
Coefficient of Kurtosis	−1.0534 (*p* = 0.0765)

**Table 3:** Logistic regression predicting treatments based on AAST scores.

Dependent Y		Treatment	
Sample size		89	
Cases with Y = 0		75 (84.27%)	
Cases with Y = 1		14 (15.73%)	
**Overall Model Fit**			
Model		Value	
Null model −2 Log Likelihood		77.46045	
Full model −2 Log Likelihood		31.69450	
Chi-square		45.7659	
DF		1	
Significance level		*p* < 0.0001	
**Coefficients and Standard Errors**			
Variable	Coefficient	Std. Error	*p*
AAST	2.3260	0.5746	0.0001
Constant	−9.2212		
**Odds Ratios and 95% Confidence Intervals**			
Variable	Odds Ratio	95% CI	
AAST	10.2373	3.3192 to 31.5745	

**Table 4:** Distribution of patients by mean arterial pressure (MAP) (mmHg).

Variable	MAP (mmHg)
Lowest value	46.6700
Highest value	139.3333
Arithmetic mean	87.8688
95% CI for the mean	84.2743 to 91.4632
Median	90.0000
95% CI for the median	86.6700 to 93.3300
Variance	291.1625
Standard deviation	17.0635
Standard error of the mean	1.8087
Coefficient of Skewness	−0.0418 (*p* = 0.8654)
Coefficient of Kurtosis	0.6620 (*p* = 0.1942)
Normality Test	Accept Normality (*p* = 0.1081)
Chi-square test for Normal Distribution	Chi-square = 16.993 (DF = 11)

**Table 5:** Distribution of patients by heart rate (bpm).

Variable	Heart Rate (bpm)
Lowest value	50.0000
Highest value	123.0000
Arithmetic mean	84.4091
95% CI for the mean	81.2668 to 87.5514
Median	85.0000
95% CI for the median	80.0000 to 89.1017
Variance	219.9457
Standard deviation	14.8306
Standard error of the mean	1.5809
Coefficient of Skewness	0.3387 (*p* = 0.1808)
Coefficient of Kurtosis	0.4153 (*p* = 0.3479)
Normality Test	Accept Normality (*p* = 0.8203)
Chi-square test for Normal Distribution	Chi-square = 6.732 (DF = 11)

**Table 6:** Distribution of patients by blood urea nitrogen levels (mg/dL).

Variable	BUN (mg/dL)
Sample size	89
Lowest value	13.0000
Highest value	136.1000
Arithmetic mean	40.0761
95% CI for the mean	35.8159 to 44.3363
Median	36.0000
95% CI for the median	32.9896 to 42.0000
Variance	409.0045
Standard deviation	20.2239
Relative standard deviation	0.5046 (50.46%)
Standard error of the mean	2.1437
Coefficient of Skewness	2.6481 (*p* < 0.0001)
Coefficient of Kurtosis	9.7086 (*p* < 0.0001)
Normality Test	Reject Normality (*p* = 0.0019)
Chi-square test for Normal Distribution	Chi-square = 24.545 (DF = 8)

**Table 7:** Distribution of patients by creatinine levels (mg/dL).

Variable	Creatinine Levels (mg/dL)
Sample size	89
Lowest value	0.53
Highest value	8.23
Arithmetic mean	1.3554
95% CI for the mean	1.0872 to 1.6236
Median	1.1000
95% CI for the median	1.0000 to 1.1373
Variance	1.6210
Standard deviation	1.2732
Relative standard deviation	0.9394 (93.94%)
Standard error of the mean	0.135
Coefficient of Skewness	3.9265 (*p* < 0.0001)
Coefficient of Kurtosis	15.8136 (*p* < 0.0001)
Normality Test	Reject Normality (*p* = 0.0001)
Chi-square test for Normal Distribution	Chi-square = 172.887 (DF = 6)

**Table 8:** Distribution of patients by hospital length of stay (days).

Statistic	Hospital Length of Stay (days)
Lowest value	1.0000
Highest value	35.0000
Arithmetic mean	8.3708
95% CI for the mean	7.0803 to 9.6613
Median	7.0000
95% CI for the median	5.0000 to 8.1038
Variance	37.5314
Standard deviation	6.1263
Standard error of the mean	0.6494
Coefficient of Skewness	1.6883 (*p* < 0.0001)
Coefficient of Kurtosis	3.7126 (*p* = 0.0003)
Chi-square test for Normal distribution	reject Normality (*p* < 0.0001)
Additional info	(Chi-square = 42.068, DF = 9)

## Data Availability

Data is unavailable due to privacy restrictions.
